# *HLA* in isolated REM sleep behavior disorder and Lewy body dementia

**DOI:** 10.1002/acn3.51841

**Published:** 2023-07-04

**Authors:** Eric Yu, Lynne Krohn, Jennifer A. Ruskey, Farnaz Asayesh, Dan Spiegelman, Zalak Shah, Ruth Chia, Isabelle Arnulf, Michele T. M. Hu, Jacques Y. Montplaisir, Jean‐François Gagnon, Alex Desautels, Yves Dauvilliers, Gian Luigi Gigli, Mariarosaria Valente, Francesco Janes, Andrea Bernardini, Birgit Högl, Ambra Stefani, Abubaker Ibrahim, Anna Heidbreder, Karel Sonka, Petr Dusek, David Kemlink, Wolfgang Oertel, Annette Janzen, Giuseppe Plazzi, Elena Antelmi, Michela Figorilli, Monica Puligheddu, Brit Mollenhauer, Claudia Trenkwalder, Friederike Sixel‐Döring, Valérie Cochen De Cock, Luigi Ferini‐Strambi, Femke Dijkstra, Mineke Viaene, Beatriz Abril, Bradley F. Boeve, Guy A. Rouleau, Ronald B. Postuma, The International LBD Genomics Consortium, Sonja W. Scholz, Ziv Gan‐Or

**Affiliations:** ^1^ Department of Human Genetics McGill University Montréal Québec Canada; ^2^ The Neuro (Montréal Neurological Institute‐Hospital) McGill University Montréal Québec Canada; ^3^ Department of Neurology and Neurosurgery McGill University Montréal Québec Canada; ^4^ Neurodegenerative Diseases Research Unit National Institute of Neurological Disorders and Stroke Bethesda Maryland USA; ^5^ Neuromuscular Diseases Research Section National Institute on Aging Bethesda Maryland USA; ^6^ Sleep Disorders Unit, Pitié Salpêtrière Hospital Paris Brain Institute and Sorbonne University Paris France; ^7^ Oxford Parkinson's Disease Centre (OPDC) University of Oxford Oxford UK; ^8^ Division of Neurology, Nuffield Department of Clinical Neurosciences University of Oxford Oxford UK; ^9^ Center for Advanced Research in Sleep Medicine Centre Intégré Universitaire de Santé et de Services Sociaux du Nord‐de‐l'Île‐de‐Montréal – Hôpital du Sacré‐Coeur de Montréal Montréal Québec Canada; ^10^ Department of Psychiatry Université de Montréal Montréal Québec Canada; ^11^ Department of Psychology Université du Québec à Montréal Montréal Québec Canada; ^12^ Department of Neurosciences Université de Montréal Montréal Québec Canada; ^13^ National Reference Center for Narcolepsy, Sleep Unit, Department of Neurology, Gui‐de‐Chauliac Hospital, CHU Montpellier University of Montpellier, Inserm U1061 Montpellier France; ^14^ Clinical Neurology Unit, Department of Neurosciences University Hospital of Udine Udine Italy; ^15^ Department of Medicine (DAME) University of Udine Udine Italy; ^16^ Sleep Disorders Clinic, Department of Neurology Medical University of Innsbruck Innsbruck Austria; ^17^ Department for Sleep Medicine and Neuromuscular disease University Hospital Muenster Muenster Germany; ^18^ Department of Neurology and Centre of Clinical Neuroscience Charles University, First Faculty of Medicine and General University Hospital Prague Czech Republic; ^19^ Department of Neurology Philipps University Marburg Germany; ^20^ Department of Biomedical, Metabolic and Neural Sciences University of Modena and Reggio‐Emilia Modena Italy; ^21^ IRCCS, Institute of Neurological Sciences of Bologna Bologna Italy; ^22^ Neurology Unit, Movement Disorders Division, Department of Neurosciences, Biomedicine and Movement Sciences University of Verona Verona Italy; ^23^ Department of Medical Sciences and Public Health, Sleep Disorder Research Center University of Cagliari Cagliari Italy; ^24^ Paracelsus‐Elena‐Klinik Kassel Germany; ^25^ Department of Neurosurgery University Medical Centre Göttingen Göttingen Germany; ^26^ Sleep and Neurology Unit Beau Soleil Clinic Montpellier France; ^27^ EuroMov Digital Health in Motion University of Montpellier IMT Mines Ales Montpellier France; ^28^ Department of Neurological Sciences Università Vita‐Salute San Raffaele Milan Italy; ^29^ Laboratory for Sleep Disorders St. Dimpna Regional Hospital Geel Belgium; ^30^ Department of Neurology St. Dimpna Regional Hospital Geel Belgium; ^31^ Department of Neurology University Hospital Antwerp Edegem Antwerp Belgium; ^32^ Sleep disorder Unit Carémeau Hospital, University Hospital of Nîmes Nîmes France; ^33^ Department of Neurology Mayo Clinic Rochester Minnesota USA; ^34^ Department of Neurology Johns Hopkins University Medical Center Baltimore Maryland USA

## Abstract

Synucleinopathies‐related disorders such as Lewy body dementia (LBD) and isolated/idiopathic REM sleep behavior disorder (iRBD) have been associated with neuroinflammation. In this study, we examined whether the human leukocyte antigen (*HLA*) locus plays a role in iRBD and LBD. In iRBD, HLA‐DRB1*11:01 was the only allele passing FDR correction (OR = 1.57, 95% CI = 1.27–1.93, *p* = 2.70e‐05). We also discovered associations between iRBD and *HLA‐DRB1* 70D (OR = 1.26, 95%CI = 1.12–1.41, *p* = 8.76e‐05), 70Q (OR = 0.81, 95%CI = 0.72–0.91, *p* = 3.65e‐04) and 71R (OR = 1.21, 95%CI = 1.08–1.35, *p* = 1.35e‐03). Position 71 (*p*_omnibus_ = 0.00102) and 70 (*p*_omnibus_ = 0.00125) were associated with iRBD. Our results suggest that the *HLA* locus may have different roles across synucleinopathies.

## Introduction

Isolated/idiopathic REM sleep behavior disorder (iRBD) is a prodromal synucleinopathy characterized by enactment of dreams, vocalization, and absence of muscle atonia during REM sleep.^1^ iRBD is one of the strongest predictors for certain neurodegenerative disorders, as approximately 80% of patients will convert to Parkinson's disease (PD), Lewy body dementia (LBD), or multiple system atrophy (MSA) after 10–15 years on average following iRBD diagnosis.^2^

Previous evidence has shown that iRBD and synucleinopathies share a partial genetic overlap.^3^ While particular loci (*SNCA*, *GBA*, and *TMEM175*) were shared between these traits, distinct loci such as *LRRK2* and *MAPT* for PD and *APOE* LBD were also identified.^3^ Furthermore, while the *SNCA* locus is essential in PD, LBD, and iRBD, the association with *SNCA* is driven by different variants for the different traits.^3^ Similar phenomenon occurs in the *SCARB2* locus, where different variants are associated with PD or RBD.^3^ Understanding the shared genes and pathways and the genetic differences will lead to better characterization of these disorders. For instance, microglial activation, a form of neuroinflammation, was found in all these disorders.^4^, ^5^ However, the immune system's role in their pathophysiology is poorly understood.

Recently, a fine‐mapping study of the human leukocyte antigen (*HLA*) locus in PD demonstrated a strong association of HLA‐DRB1 amino acids 11 V, 13H, and 33H with reduced PD risk.^6^ Located on chromosome 6, the *HLA* locus is a highly polymorphic region with complicated linkage patterns. *HLA* plays an essential role in the adaptive immune system by presenting antigens to T cells.

Since the role of the *HLA* locus is unknown in iRBD and LBD, this study aims to examine whether *HLA* variants may affect the risk for these disorders. We analyzed the association of different *HLA* alleles, haplotypes, and amino acids in two cohorts of iRBD and LBD patients.

## Methods

### Study population

iRBD and LBD cohorts from two previous genome‐wide association studies (GWAS) were included in this analysis (Table 1).^3^, ^7^ iRBD patients were diagnosed according to the International Classification of Sleep Disorders (2nd or 3rd Edition) with video polysomnography. LBD was diagnosed according to consensus criteria, as described elsewhere.^7^ The LBD cohort was not screened for iRBD. The iRBD cohort is composed of 1072 patients and 9505 controls with genotyping data from the OmniExpress GWAS chip (Illumina Inc.). The control group includes six publicly available cohorts: controls from the International Parkinson's Disease Genomics Consortium (IPDGC) NeuroX dataset (dbGap phs000918.v1.p1), National Institute of Neurological Disorders and Stroke (NINDS) Genome‐Wide genotyping in Parkinson's Disease (dbGap phs000089.v4.p2), NeuroGenetics Research Consortium (NGRC) (dbGap phs000196.v3.p1), Parkinson's Progression Markers Initiative (PPMI), and Vance (dbGap phs000394).

**Table 1:** Study population after quality control.

Variable	Isolated REM sleep behavior disorder		Lewy body dementia	
	Patients (*n* = 1072)	Controls (*n* = 9505)	Patients (*n* = 2604)	Controls (*n* = 4032)
Age (years), (SD)	60.54 (11.06)	63.49 (16.59)	74.36 (11.76)	72.63 (16.99)
Male, number (%)	860 (80.22)	4824 (50.75)	1656 (63.59)	1967 (48.78)

*n*, number; SD, standard deviation.

The LBD cohort consisted of 2604 patients and 4032 controls with whole‐genome sequencing data as described elsewhere.^7^ Study participants signed informed consent forms and the Institutional Review Board at McGill University approved the study protocol.

### Quality control

We performed standard GWAS quality control steps for both cohorts using PLINK v1.90. We excluded variants that were heterozygosity outliers (|F| > 0.15), sample call rate outliers (<0.95) and samples failing sex checks were also excluded. We determined genetic ancestry by merging samples with HapMap3 and clustering with principal components analysis (PCA). We only selected samples of European ancestry. A relatedness check was performed with GCTA to remove third‐degree relatives or closer ones. Then, we performed several variant‐level filtrations, such as removing call rate outliers (<0.95) and variants with significantly different missingness between cases and controls (*p* < 0.0001). We also excluded variants that failed PLINK –test‐mishap (*p* < 0.0001) and deviated from Hardy–Weinberg equilibrium (*p* < 0.0001) in controls.

### HLA imputation

Samples passing quality control were imputed on the Michigan Imputation Server with the four‐digit multiethnic HLA reference panel v2^8^ using Minimac4 and phased with Eagle v2.4. This reference panel is composed of five global populations (*n* = 20,349). Only alleles with an imputation score (R^2^) above 0.8 were included. We determined *HLA* haplotypes using haplo.stats R package (https://analytictools.mayo.edu/research/haplo‐stats/), which employs an Expectation–maximization (EM) algorithm. Using the HLA Genotype Imputation with Attribute Bagging (HIBAG) R package, we imputed HLA amino acids from HLA alleles.^9^

### Power calculations

We performed power calculations online for each cohort using CaTS to compute statistical power. (https://csg.sph.umich.edu/abecasis/gas_power_calculator/). We assumed a prevalence of 1% for iRBD^10^ and LBD.^11^ We had enough statistical power (>0.8) to detect an association (*p* = 0.0005) with an odds ratio of 1.33 with a minor allele frequency (MAF) of 0.15. We chose a MAF of 0.15, which correspond to the frequency of *HLA*‐*DRB1* 33H, the amino acid associated with PD. However, we had less than 80% statistical power to detect this association.

### Statistical analysis

We performed logistic regression with an additive model on each *HLA* allele, haplotype and amino acid after adjusting for age at onset, sex and the top 10 principal components. To test which amino acid position is the most strongly associated with disease risk, we also performed an Omnibus test using the OMNIBUS_LOGISTIC module from HLA‐TAPAS.^8^ All rare associations (carrier frequency <1%) were excluded. A 5% false discovery rate (FDR) for multiple testing was applied for alleles (*n* = 102), haplotypes (*n* = 105), and amino acids (*n* = 716) separately.

## Results

After *HLA* imputation, we examined the association of *HLA* alleles, haplotypes, and amino acids. HLA‐DRB1*11:01 was the only allele passing FDR correction (OR = 1.57, 95% CI = 1.27–1.93, *p* = 0.00275, Table 2). In addition, *HLA‐DRB1* 70D, an amino acid encoded by DRB1*11:01, was associated with iRBD (OR = 1.26, 95%CI = 1.12–1.41, *p* = 0.0209). We also found association with 70Q (OR = 0.81, 95% CI = 0.72–0.91, *p* = 0.0441) and 71R (OR = 1.21, 95% CI = 1.08–1.35, *p* = 0.0441). In *HLA‐DRB1*, positions 71 (*p*_omnibus_ = 0.00102) and 70 (*p*_omnibus_ = 0.00125) were the most associated with iRBD. DRB1*11:01 also tags three haplotypes: DQA1*05:01–DQB1*03:01–DRB1*11:01 (OR = 1.40, 95% CI = 1.16–1.70, *p* = 0.0285), DQA1*05:01–DRB1*11:01 (OR = 1.41, 95% CI = 1.16–1.72, *p* = 0.0285), and DQB1*03:01–DRB1*11:01 (OR = 1.36, 95% CI = 1.13–1.64, *p* =0.0364).

**Table 2:** HLA association in isolated REM sleep behavior disorder.

	MAF in cases	MAF in controls	OR	95% CI	*p*	*p* (FDR)
Alleles						
HLA‐DRB1*11:01	0.0726	0.0472	1.57	1.27–1.93	2.70e‐05	2.75e‐03
Amino acids						
*HLA‐DRB1* 70D	0.505	0.444	1.26	1.12–1.41	8.76e‐05	2.09e‐02
*HLA‐DRB1* 70Q	0.440	0.503	0.81	0.72–0.91	3.65e‐04	4.41e‐02
*HLA‐DRB1* 71R	0.545	0.496	1.21	1.08–1.35	1.35e‐03	4.41e‐02
Haplotype						
DQA1*05:01–DQB1*03:01–DRB1*11:01	0.0924	0.0657	1.40	1.16–1.70	5.17e‐04	2.85e‐02
DQA1*05:01–DRB1*11:01	0.0933	0.0652	1.41	1.16–1.72	5.43e‐04	2.85e‐02
DQB1*03:01–DRB1*11:01	0.0989	0.0707	1.36	1.13–1.64	1.04e‐03	3.64e‐02

CI, confidence interval; FDR, false discovery rate for each group; MAF, minor allele frequency; OR, odds ratio; *p*, *p*‐value.

When we repeated the analysis at one‐field (two‐digit) resolution, for example, treating DRB1*11:01 and 11:04 as the same, the association of DRB1*11 was not significant (*p* = 0.12, Table S1), suggesting that the association was driven solely by the DRB1*11:01 allele. For LBD, no association was statistically significant after correction for multiple comparisons. We also examined the association of *HLA‐DRB1* 33H, which was previously reported to be associated with PD (Table S3).^6^ Although DRB1 33H was not associated with iRBD (*p* = 0.84), the MAF frequencies in cases and controls were 0.125 vs. 0.149, respectively. Meanwhile, the DRB1 33H allele frequency in both LBD cases and its controls was 0.145 (Table S7). Our results suggest that DRB1 33H could be associated with iRBD, but our study lacks the power to detect it.

## Discussion

This study shows an association between DRB1*11:01, DRB1 70D, 70Q, and 71R on iRBD. We also identified HLA‐DRB1 positions 71 and 70 via an omnibus test, which suggests that residues at those positions explain a large amount of variance. HLA‐DRB1 position 70–74 is a strong risk factor for rheumatoid arthritis and is referred to as a “shared epitope” (SE).^12^ The SE, in combination with DRB1 11 V, was associated with a protective effect for PD.^13^ The SE is composed of 70 Q/R and 71 K/R, which are important antigen‐binding grooves. Additional studies examining how the SE mediates risk for iRBD will be necessary.

In addition, DRB1 33H, a variant also associated with PD, was not significantly associated with iRBD or LBD. However, the difference in carrier frequency between iRBD cases and controls for DRB1 33H, similar to that seen in PD, suggests that our study may lack the power to detect this association in iRBD. A recent study has suggested a shared mechanism between PD, AD, amyotrophic lateral sclerosis and HLA‐DRB1*04, harboring the 33H amino acid change.^14^ This subtype was associated with decreased neurofibrillary tangles in post‐mortem brains. It also binds to a K311 acetylated Tau PHF6 sequence.^14^ These results exemplify the possibility of different HLA types with specific genetic variants that may affect the binding of substrates relevant for neurodegenerative disorders and activating inflammatory response.

We could not replicate the association of a previous study of HLA antigens with 25 iRBD cases. This study showed a significant association between iRBD and DQB1*05 and DQB1*06.^15^ The most likely explanation for the discrepancy is that the previous study lacked the power to detect an actual effect. Another study has suggested that HLA‐DR expression was associated with iRBD.^16^ Fine‐mapping and colocalization studies for these findings will be required once larger datasets of iRBD become available. Whether the mechanism underlying the associations with PD and iRBD is through functional effects of specific amino acid changes or due to different expressions of *HLA* genes in various brain tissues is still to be determined.

Although the immune system's involvement is still elusive, some potential mechanisms of effect may exist. The varying effects of HLA between RBD and PD could be associated with changing T‐cell responses during disease progression. One study showed that alpha‐synuclein‐specific T‐cell responses are high before the development of PD motor symptoms and decline after.^17^

Another possibility is that the varying effects between iRBD and PD originate from the brain‐first or body‐first hypothesis.^18^ This model hypothesizes that in brain‐first PD, alpha‐synuclein originates from the brain and spreads to the peripheral autonomic nervous system. In body‐first PD, pathology originates in the gut and spreads to the brain. RBD‐positive and negative PD cases were associated with gut‐first and brain‐first, respectively. HLA alleles may induce a different immune response to brain‐first and body‐first diseases. Additional mechanisms, which have not been discovered, may be responsible for the association.

Our study has several limitations. First, future replication studies with larger cohorts would be needed to increase statistical power since we do not have a replication cohort. Note that we used the most extensive available cohorts for iRBD and LBD.^3^, ^7^ Although the LBD cohort was composed of Parkinson's disease with dementia (PDD) and dementia with Lewy bodies (DLB) patients, PDD represents only 145 out of 2604 LBD cases. The PDD samples may likely have a minimal effect on the results. Due to the polygenicity of the HLA locus, various populations have different HLA allele frequencies. This study was done only on samples with European ancestry, and multiancestry analysis could provide more refined evidence on the role of HLA in synucleinopathies. The cohorts used in the study were also not matched for age and sex. However, we adjusted for these variables in the analysis.

To conclude, we found an alternative *HLA* association of iRBD compared with PD and LBD. More experimental evidence is necessary to characterize the genetic landscape of synucleinopathies and the immune system's role.

## Author Contributions

E.Y. and Z.G.O. contributed to conception and design of the study; E.Y, L.K., J.A.R., F.A., D.S., Z.S., R.C., I.A., M.T.M.H., J.Y.M., J‐F.G., A.D., Y.D., G.L.G., M.V., F.J., A.B., B.H., A.S., A.I., A.H., K.S., P.D., D.K., W.O., A.J., G.P., E.A., M.F., M.P., B.M., C.T., F.S.D., V.C.D.C, C.C.M., L.F.S., F.D., M.V., B.A., B.F.B, G.A.R., R.B.P., ILBDGC, and S.W.S contributed to the acquisition and analysis of data; E.Y. and Z.G.O. contributed to drafting of the manuscript. A full list of ILBDGC members is listed in Table S8.

## Conflict of Interest

S.W.S. serves on the Scientific Advisory Council of the Lewy Body Dementia Association and the Multiple System Atrophy Coalition. S.W.S. receives research support from Cerevel Therapeutics.

## Code Availability Statement

All scripts used in this study can be found at https://github.com/gan-orlab/HLA_syn.

## Supporting information


Tables S1–S7

Table S8

## Data Availability

Anonymized data not published within this article will be made available by request from any qualified investigator.
